# Demographic Factors Influencing Consensus Opinion on the Recall for Women Screened by Mobile Mammography Unit in Taiwan

**DOI:** 10.5812/iranjradiol.6952

**Published:** 2013-08-30

**Authors:** Lee Yu-Mei, Yao Hsueh-Hua

**Affiliations:** 1 Health and Management Center, Cardinal Tien Hospital, Xindian District, New Taipei City, Taiwan, Republic of China; 2 Department of Medical Imaging and Radiological Technology, Yuanpei University, Hsinchu City, Taiwan, Republic of China

**Keywords:** Breast Neoplasms, Mammography, Breast

## Abstract

**Background:**

The incidence of breast cancer has had a four-fold increase from 1980 to 2005 in Taiwan. Limited data have been available on mobile breast screening in the Taiwanese population since 2009.

**Objectives:**

This study aims at investigating the factors influencing consensus opinion on the recall for mobile breast screening in Taiwan.

**Patients and Methods:**

The factors were categorized by individual health background, socioeconomic status and knowledge about breast screening. There were 502 questionnaires collected from Taiwanese women examined on mobile mammography screening vehicle. Data were then analyzed by SPSS 12 via analysis of variance (ANOVA), F-test, t-test or chi-square test.

**Results:**

Strong participation was associated with a younger age, higher educational level, higher incomes, previous history of cancer, previous family history of cancer, one or two prior mammographies, more correct recognitions of mammography, recall rate, and breast cancer risk. If the false-positive result occurred, 83.9%, 81.9% and 77.3% of the women agreed or strongly agreed to participate in noninvasive and invasive testing and screening mammography, respectively.

**Conclusion:**

The policy makers should notify the importance of demographic factors affecting further examination for early detection of breast cancer in Taiwan.

## 1. Background

Screening mammography has been demonstrated to decrease breast cancer mortality by approximately 30% (1). In Taiwan, the incidence of breast cancer has had a four-fold increase from 1980 to 2005 (49 per 100,000 women in 2005) (2). From Oct 2002 to May 2005, the breast cancer detection rate of Taiwanese women aged 50 to 69 years who received two-way screening mammography has been reported as 4% (3). Since 2009, mobile breast screening facility has been introduced and has influenced the breast screening service. For example, Ho et al. (2) reported that the mobile breast screening service has significantly increased the volume of their breast screening, but it has helped the work of their breast cancer treatment team a little. In addition, the mobile breast screening service somehow resulted in a trade-off with their “in hospital" breast screening practice causing reduction of volume of mammography in the hospital. The outpatient department recall rate is significantly lower compared to the “in hospital" breast screening group.

There were many studies addressing the parameters related to desire for recall and willingness to continue with annual screening mammography once given a false-positive result in hospital. Tatla et al. (4) found that not only should urban women of lower socioeconomic status be specifically targeted to come in for an initial screen, but also programs may also need to focus on retention strategies following the prevalence screening. The study conducted by Ganott et al. (5) showed that the majority (97%) of women in the study group were white and thought that a false-positive test would not deter them from continuing screening mammography and they strongly preferred a higher rate of recall to undergo both invasive and noninvasive testing if it translated into chance of earlier detection . The study by Jafri et al. (6) reported that differences in ethnic background, i.e., white, black, and Hispanic, appeared to influence women’s understanding of mammography, compliance with recall, and preference for early detection of breast cancer. Pornet et al. (7) described that even with organized breast cancer screening giving screening free of charge for target women, ecological socioeconomic factors had a more significant impact on participation than healthcare supply. Kinnear et al. (8) suggested that changes that would result in increasing the uptake in cities may help reduce socio-economic inequalities in cancer screening.

## 2. Objectives

Due to the lower incidence rate of breast cancer in the Chinese population and hence issues with cost effectiveness, population-based screening is still rather controversial. There are limited data available on breast screening in the Taiwanese population especially that of those screened by mobile mammography vehicle. The purpose of our study was to prospectively survey the opinion and preferences of Taiwanese women screened by mobile mammography vehicle regarding their understanding of screening mammography, their desire for recall and early detection, and their willingness to continue with annual screening mammography once given a false-positive result.

## 3. Patients and Methods

From November 2011 to February 2012, a total of 502 women arriving at two different breast screening vehicles in Taiwan were asked to complete the questionnaire of an Institutional Review Board-approved survey conforming to the ethical guidelines of the 1975 Declaration of Helsinki that was in Chinese, anonymous, and strictly voluntary. Informed consent was obtained from each person included in the study. The questionnaire was designed to find whether individual health background, socioeconomic status, and knowledge about breast screening affect the desire for recall. The validity of all the 17 questions was evaluated by 5 radiological experts in the form of content validity index (CVI) and if needed, the internal consistency was measured by Cronbach's alpha. The questionnaire consisted of three major parts. Part I elicited demographic information, such as age, highest education level, annual household income, family or personal history of breast cancer, and number of prior screening mammograms. Part II of the questionnaire assessed adherence to recall after positive-false study and asked women to identify their best estimate of the sensitivity of mammography for the detection of breast cancers, their best estimate of the current recall rate, their risk of breast cancer, their relative chance of cancer detection with one mammography, and their preference for further invasive and noninvasive testing. A five-point Likert scale including (1) strongly disagree, (2) disagree, (3) not sure about it, (4) agree, or (5) strongly agree, was adopted to specify their preference for recall for invasive and noninvasive testing. Part III of the questionnaire included three factors that might influence the recall preference: the unit that notified this screening test, the preference and reason for further mammography in the screening vehicle or in hospital.

Depending on the variable type and the comparison type, F-test, t-test, chi-square test, or analysis of variance (ANOVA) were used to determine the significance of the preference for recall for invasive and noninvasive testing. P value of less than 0.05 was considered to indicate a statistically significant difference for all comparisons. The Duncan’s test was used to give information about significant differences among groups in the multiple comparisons.

## 4. Results

To ensure that the 17 questions of the questionnaire were measured validly and reliably, average CVI and Cronbach's alpha were determined respectively. The questionnaire was demonstrated a good content validity by having a CVI of 0.962. In addition, a Cronbach's alpha of 0.915 confirmed high reliability of the questionnaire as this value is excellent considering that 0.70 is the cutoff value for an acceptable reliability.

Of the 502 women, the majority of participants (298 of 502, 59.4%) were aged 51-60 years (Table 1). The willingness for further noninvasive testing was lower among the older (>50 years) compared to the younger group (<50 years) (F=21.6, P<0.001). The 46-50 year age group reported a higher preference to participate invasive testing (F=23.8, P<0.001). There were significant differences for continuing mammographic study among age subgroups (F=22.5 and P<0.001. The allowance order was the youngest (≤50 years), the intermediate (51-60 years), and the oldest (>60 years). 

**Table 1.** Socioeconomic Characteristics of 502 Women

Parameter	No. of Respondents, (%)
**Age (y)**	
40-44	4 (0.8)
45-50	107 (21.3)
51-55	151 (30.1)
56-60	147 (29.3)
>60	93 (18.5)
**Highest education level**	
Elementary school	129 (25.7)
Junior high school	180 (35.9)
Senior high school	108 (21.5)
Junior college diploma	37 (7.4)
College degree	46 (9.2)
Graduate degree	2 (0.4)
**Annual Household Income (TWD$, million)**	
<0.3	131 (26.1)
0.3 - 0.6	194 (38.6)
0.6 - 0.9	120 (23.9)
0.9 - 1.2	52 (10.4)
>1.2	5 (1)

One hundred and eighty (35.9%) of 502 women responded as a graduate from a junior college (Table 1). The willingness for noninvasive testing was higher among the higher education level group compared to the lower group (F=42.1, P<0.001). Similarly, the higher education level group reported a higher likelihood to participate invasive testing (F=43.5, P<0.001) and continuing with mammography (F=39.6, P<0.001). 

A percentage of 38.6 (194 of 502) reported an annual household income of TWD$ 60 k-90 k (Table 1). The willingness for noninvasive testing was higher among the higher annual household income group compared to the lower group (F=42.4, P<0.001). Comparably, the higher group reported a higher likelihood to participate invasive testing (F=44.2, P<0.001) and continuing with a mammographic study (F=32.4, P<0.001). 

Most participants did not respond a personal (477 of 502, 95%) or family (386 of 502, 76.9%) history of breast cancer (Table 2). The majority of participants (397 of 502, 79.1%) had undergone one or more screening mammograms previously. The preference for noninvasive testing, invasive testing, and continuing with mammography was higher among the women who had a personal or family history of breast cancer (P<0.001). Women who reported undergoing one or two prior screening mammograms were more likely to continue with screening mammography than those reporting none and three or more prior screening mammograms in the future (P<0.001). 

**Table 2.** Health Background Characteristics of 502 Women

Parameter	No. of Respondents, (%)
**Personal history of chronic disease**	
High blood pressure	61 (12.2)
Diabetes	21 (4.2)
Heart disease	11 (2.2)
Hyperlipidemia	48 (9.5)
Kidney disease	1 (0.2)
None	377 (75.1)
**Personal history of breast cancer**	
Yes	25 (5)
No	477 (95)
**Family history of breast cancer**	
Yes	116 (23.1)
No	386 (76.9)
**Number of prior screening mammograms**	
None	105 (20.9)
1	165 (32.9)
2	147 (29.3)
≥3	85 (18.9)

After receiving false-positive results, 83.9% (421 of 502) and 81.9% (411 of 502) of the women agreed or strongly agreed to participate noninvasive and invasive testing, respectively (Table 3). A percentage of 77.3 (388 of 502) stated likely or very likely to continue with screening mammography in the future. 

Sixty-nine percent (345 of 502) of the women identified they might be recalled for additional tests after a screening mammogram (Table 4). In the literature, it has been demonstrated that mammography exhibited a highly sensitive test that enables detection of 77.8%-95% of breast cancers (9, 10). Forty-two percent (212 of 502) of the women correctly identified the sensitivity of mammography (80%-94%) for breast cancer. The recall rate of mammography was reported 12% (6) and 12.2% (11). Sixty-seven percent (336 of 502) of the women correctly identified the recall rate of mammography (10% and 15%) for breast cancer. Most women (63.9%, 321 of 502) believed that their general breast cancer risk was approximately the same as that of most women. Moreover, we found that respondents who accurately identified the current detection rate and recall rate were more likely to continue with noninvasive and invasive testing and screening mammography (P<0.001). 

**Table 3.** Preference for Further Testing (n=502)

Parameter	No. of Respondents, (%)
**Preference for further noninvasive testing**	
Disagree	1 (0.2)
Not sure	80 (15.9)
Agree	301 (60.0)
Strongly agree	120 (23.9)
**Preference for further invasive testing**	
Disagree	0 (0)
Not sure	91 (18.1)
Agree	300 (59.8)
Strongly agree	111 (22.1)
**Preference for further mammographic screening**	
Hesitant	1 (0.2)
Not sure	113 (22.5)
Likely to continue	244 (48.6)
Very likely to continue	144 (28.7)

**Table 4.** Cognition Related to Screening Mammogram and Recall (n=502)

Question and answer	No. of Respondents, (%)
**Do you think you might be recalled for additional tests after a screening mammogram?**	
Yes	345 (68.7)
No	157 (31.3)
**How many breast cancers do you think mammography can detect?**	
100%	28 (5.6)
95%	92 (18.3)
80%-94%	212 (42.2)
50%-79%	111 (22.1)
<50%	59 (11.8)
**Approximately how many women do you think are currently being recalled for additional tests after a screening mammogram?**	
1%	26 (6.2)
5%	65 (12.9)
10%	227 (45.2)
15%	109 (21.7)
20%	56 (11.2)
30%	19 (3.8)
**What do you think is your risk of having a breast cancer detected on this screening mammogram compared to an average woman’s risk of having breast cancer detected?**	
The same	321 (63.9)
Lower	130 (25.9)
Higher	51 (10.2)

**Table 5.** Participation Information About the Mammographic Screening (n = 502)

Question and answer	No. of Respondents, (%)
**How did you know the notification to mammography screening?**	
Television	11 (2.2)
Health center	344 (68.5)
Hospital	146 (29.1)
Network	11 (2.2)
Friends or relatives	6 (1.2)
**Preference for location of further mammography**	
Mammography screening bus	107 (21.3)
Hospital	38 (7.6)
Both	357 (71.1)
**Reasons for choosing the screening location**	
Convenience	265 (53.4)
Sense of security	40 (8.1)
Reputation of hospital	45 (9.1)
Grade of hospital	61 (12.3)
Sense of comfort	106 (21.4)

The final part of the survey included probable parameters influencing the recall preference. We found that the majority of participants (68.5%, 344 of 502) were notified to mammography by the regional health center. Most women (71.1%, 357 of 502) thought that both the mobile vehicle and hospital were similar for mammography in the future. However, 21.3% women preferred mobile vehicle for the screening compared to 7.6% of hospital. While participants chose a screening location, convenience (53.4%, 265 of 502) and sense of comfort (21.4%) were the important considerations (Table 5). 

## 5. Discussion

Our main objective was to determine the preferences of the Taiwanese women who had undergone mammography in mobile mammography vehicles for recall and the potential for earlier detection of cancer, as well as willingness to continue with future testing once given false-positive results. Similar to the some other studies (5, 6), most women (42.2%) in our study had a general understanding of the sensitivity of screening mammography, were likely to continue with screening despite false-positive results (77.3%), and preferred noninvasive and invasive testing (83.9% and 81.9%, respectively) if it meant earlier detection of cancer. However, it should be noted that there was a self-selection bias in this study population, as the respondents who were screened in a mobile mammography vehicle in Taiwan had a low annual income and education level, and women screened in the hospital were underrepresented. We would seek to address this limitation and improve the survey results to a broader and more diverse patient population in the future.

The results of our study agreed with the results of previous studies in which women who had undergone screening examinations in the past were more likely to continue with annual screening than those who had not undergone a previous screening examination (5). Interestingly, women who had undergone at least three prior screening mammographic examinations were less likely than women who were undergoing their initial or less screening examination to continue with screening and noninvasive and invasive testing (P<0.001). This finding might be the result of women fearing the consequences and/or the discomfort of mammography.

Women in a study most frequently cited fear of results as the main deterrent to adherence to recall (6). These women might have been less likely to prefer an increased recall rate because of a limited understanding of mammography and possibly because of the belief that breast cancer is an untreatable illness. Our study specifically elicited the underlying reasons beneath these preferences and determined the forums in which our patients are being educated about the use of mammography or breast cancer. We suggested that regional health center and hospital should educate populations more. Besides, improving the convenience and sense of comfort in mammographic screening would be chief concerns.

Much of the analysis provided in this study was related to several demographic factors, such as income and education level, and their association with a patient’s adherence to screening mammography and recall; however, our study was not powered with a sufficient sample size to allow a stratified analysis of over more than one variable simultaneously. This would be an important issue to investigate in further work.

Many studies (5, 6) noted self-selection biases, which might have been similarly present in our study. It was unclear whether the population of women undergoing screening mammography in our study was identical to the general medical center population. Similar to all voluntary surveys, there is a higher likelihood of participation of women who are currently joining in routine annual screening. Additionally, analysis of the participants’ answers to estimation of recall is challenging, as recall rates vary widely across institutions and populations (12-14).
